# Profiles of Serum Fatty Acids in Healthy Women on Different Types of Vegetarian Diets

**DOI:** 10.3390/nu16040516

**Published:** 2024-02-13

**Authors:** Patrycja Gogga, Adriana Mika, Agata Janczy, Alicja Sztendel, Tomasz Sledzinski, Sylwia Małgorzewicz

**Affiliations:** 1 Department of Food Commodity Science, Medical University of Gdansk, ul. Debinki 7, 80-211 Gdansk, Poland; patrycja.gogga@gumed.edu.pl; 2 Department of Pharmaceutical Biochemistry, Medical University of Gdansk, ul. Debinki 1, 80-211 Gdansk, Poland; adriana.mika@gumed.edu.pl (A.M.); alicja.sztendel@gumed.edu.pl (A.S.); tomasz.sledzinski@gumed.edu.pl (T.S.); 3 Department of Environmental Analysis, Faculty of Chemistry, University of Gdansk, ul. Wita Stwosza 63, 80-308 Gdansk, Poland; 4 Department of Clinical Nutrition, Medical University of Gdansk, ul. Debinki 7, 80-211 Gdansk, Poland

**Keywords:** vegan diet, vegetarian diet, fatty acid profile, EPA and DHA, *n*-6 PUFAs, *n*-3 PUFAs, inflammation

## Abstract

Background: Plant-based dietary patterns are a source of different amounts and proportions of fatty acids (FAs) from those in traditional diets. Information about the full FAs profile provided by plant-based diets is widely lacking. The aim of this study was to present the exact serum profiles of FAs among people on a plant-based diet compared with omnivorous subjects. Methods: FAs compositions and inflammation statuses (based on serum C-reactive protein (CRP) levels) were studied in serum samples obtained from 102 female volunteers (divided into four groups: vegans, vegetarians, pescatarians, and omnivores). The quality of the volunteers’ diets was assessed based on seven-day dietary records. Results: Both vegans and vegetarians had lower total *n*-3 PUFAs, EPA, and DHA serum levels than omnivores. Decreased levels of these FAs presumably did not cause inflammation in vegetarians and vegans, as vegetarians had similar serum levels of CRP compared to omnivores, and vegans had even lower levels. Conclusion: The analysis of serum FAs and CRP levels in vegetarians and vegans suggests that factors other than diet alone influence inflammation and overall health status. Further research on long-term plant-based diet users is needed to better understand this issue, and supplementation with EPA and DHA is worth considering in vegans and vegetarians.

## 1. Introduction

Fatty acids (FAs) are hydrophobic molecules, which serve mainly as an important energy source for animal cells; however, some groups of these compounds play additional roles in the human body. For instance, it is well-known that *n*-3 polyunsaturated fatty acids (*n*-3 PUFAs) have anti-inflammatory and antioxidant properties, especially eicosapentaenoic acid (EPA) and docosahexaenoic acid (DHA) [1]. Additionally, there is growing evidence that other FAs perform pro-health activities as well. Odd-chain saturated FAs (OCFAs) are known for their antibiotic, anti-inflammatory, and anti-carcinogenic effects [2] and branched-chain saturated FAs (BCFAs) act as anti-dyslipidemic and anti-inflammatory agents [3], with possible anti-carcinogenic properties [2].

FA profiles in serum and cells depend on their intake and metabolism, i.e., synthesis and catabolism (β-oxidation). Plant-based diets are not only a source of different amounts and proportions of FAs from typical diets but also affect BMI and fat mass, thus having an impact on the biosynthesis of endogenic FAs as well. Therefore, it seemed interesting to investigate FAs profiles in people on different types of plant-based diets, as they have an impact on human health. Moreover, there is a lack of papers on this subject, and the research conducted so far has commonly evaluated mainly major groups of FAs, i.e., saturated fatty acids (SFAs), monounsaturated fatty acids (MUFAs), and polyunsaturated fatty acids (PUFAs), with special regard to essential FAs (e.g., linoleic acid (LA)), α-linolenic acid (ALA), EPA, and DHA [4,5,6]. The aim of this paper was to thoroughly investigate serum FAs profiles in healthy female subjects from different diet groups: two types of vegetarian diets, a pescatarian diet, and an omnivorous diet (controls).

## 2. Materials and Methods

### 2.1. Diet Types and Anthropometric Data

This study received the approval of the local Independent Bioethics Commission for Research (no: NKBBN/234/2016). It was part of the following project: “The influence of meat-free diet on serum levels of leptin and obesity markers, and gut microbiome in vegetarian and vegan subjects” (no: 01-0293/08/316). Among all participants in the project, only young women were chosen for this study to unify the study group, as there were too few men for reliable statistical analysis and sex comparisons. Eventually, 102 female volunteers entered this study: 30 vegans, 28 lacto-ovo vegetarians (referred to as vegetarians), 13 pescatarians, and 31 omnivores. The vegans’ diet lacked all animal-derived products. The vegetarians’ diet excluded meat and fish, while participants consumed dairy, eggs, and honey. The dietary pattern of the pescatarians was similar to this; however, they regularly consumed fish. The omnivores followed a typical diet, consisting of both plant and animal products, with meat eaten at least several times a week.

All women included in this study were apparently healthy and had a BMI within a normal range (18.50–24.99 kg/m^2^). Table 1 presents the basic characteristics of the studied groups. Participants were recruited into this study via electronic media. Information about the purpose and course of this research was released on Facebook, with instructions on how to join in. The inclusion criteria for the study groups were as follows: having a particular diet for at least three months continuously, absence of eating disorders, and not being pregnant or lactating. The omnivores were supposed to consume meat regularly and meet the same additional criteria as the study groups.

All women participated voluntarily in the projects and provided informed consent prior to the onset of this study. They underwent two consultations with a well-trained, experienced interviewer. During the first one, they provided detailed information about their health status and the specificity and longevity of their diets. The following anthropometric parameters were measured in all participants:

- Body mass (Jawon Medical X-contact 350, Jawon, Seongnam, Republic of Korea) and height (stadiometer Seca 213, Germany), used to calculate the body mass index (BMI) value;
- The circumference of the waist and hips (tape measure Seca 201, Seca, Hamburg, Germany), used to calculate the waist-to-hip ratio (WHR);
- Body composition (body fat content), estimated with the BIA method (Jawon Medical X-contact 350, Jawon, Seongnam, Republic of Korea).

In order to estimate nutrient intake, particularly FAs, participants completed dietary records for seven days (five working days and a weekend). During the first consultation, respondents were given detailed instructions on how to record their food intake. Respondents were supposed to precisely record every food and drink they consumed. Both the International System of Units (grams, liters, etc.) and kitchen measurements (cups, tablespoons, etc.) were allowed. During the second consultation, the interviewer verified the prepared records and, if necessary, questioned the responders about some details that were missing, e.g., consumed snacks or an accurate description of how a meal was prepared. A total of 94 out of 102 participants completed the records of food intake. The data collected from dietary records were used to estimate the intake of energy and selected dietary components, which was calculated in Aliant 2.0 (Anmarsoft, Gdańsk, Poland).

### 2.2. Sample Collection and Lipid Analysis

Peripheral venous blood was collected from subjects during the second consultation after an overnight fast and centrifugated to separate the serum, which was stored at −80 °C before the analysis. The extraction of total lipids from serum samples was performed using a chloroform–methanol mixture (2:1, *v*/*v*) based on the methodology of Folch et al. [7]. The obtained lipid extracts were dried under a nitrogen stream, reconstituted in 1 mL of 0.5 M KOH in methanol, and subjected to hydrolysis at 90 °C. After 3 h, the mixture was acidified with 0.5 mL of 6 M HCl. After this step, 1 mL of H_2_O was added to the mixture, and FAs were extracted with 3 volumes of 1 mL of n-hexane and then converted into fatty acid methyl esters (FAMEs) by incubation with a boron trifluoride–methanol solution at 55 °C for 90 min. An amount of 1 mL of H_2_O was then added to the mixture, and FAMEs were extracted with 3 volumes of 1 mL of n-hexane. Subsequently, the solvent was evaporated under a nitrogen stream, and the samples were stored at −20 °C until the analysis. The FAMEs were analyzed by GC-MS QP-2010 SE (Shimadzu, Kyoto, Japan) immediately after reconstitution in dichloromethane. The separation was carried out in a Zebron ZB-5MSi capillary column (with a 30 m length; 0.25 mm i.d.; and 0.25 m film thickness). Overall, the run time of the analysis was 60 min, with the column temperature set between 60 and 300 °C (4 °C/min). Helium was used as a carrier gas, and the head pressure in the column was 100 kPa. The electron impact source for mass detection was operated at 70 eV. The mass spectra acquisition was carried out in full-scan mode (*m*/*z* 45–700). As an internal standard, 19-methylarachidic acid was used. FAs were identified using reference standards (37 FAME Mix, Sigma-Aldrich, Saint Louis, MO, USA) and the reference library NIST 2011. C-reactive protein (CRP) concentrations were determined in serum with an XL-100 analyzer (Erba Diagnostics Mannheim GmbH, Mannheim, Germany).

### 2.3. Statistical Analysis

All basic characteristics, comparisons between diet types, and correlations were calculated in Statistica 13.3 (Statsoft). Both serum FAs levels and FAs intake were compared using parametric analysis of variance (ANOVA), with Duncan’s test applied post hoc. In three cases (*n*-3 PUFAs, EPA + DHA serum levels, and the intake of energy from macronutrients), the distributions were not normal according to the Shapiro–Wilk test; thus, the Kruskal–Wallis test was used, with Dunn’s test applied post hoc. The same tests were used to compare the CRP serum levels between diet groups, as the distribution of this variable was not normal.

To examine the differences in ALA intake and serum DHA concentrations between groups, Pearson’s correlation coefficient was used. The same coefficient was used for comparisons between the serum levels of FAs and their intake, except for *n*-3 PUFAs and EPA + DHA. Because of the non-normal distributions, Spearman’s rank correlation coefficient was chosen.

The assessment of outliers was based on boxplots. Values were excluded from general analyses if they exceeded a 1.5 interquartile deviation. In all analyses, *p* < 0.05 was considered statistically significant.

Principal component analysis (PCA) and heatmaps were performed in an open-source software, Python 3.8.18 (Python Software Foundation License), using the following libraries: Pandas, Seaborn, NumPy, SciPy, Matplotlib, and scikit-learn.

## 3. Results

We observed statistically significant differences in the serum levels of many analyzed FAs between the diet types. The only group of FAs with no differences between diets was MUFAs (Table 2). SFAs concentrations were lower in vegans compared with vegetarians and omnivores. Many differences were observed in OCFAs levels, with the lowest concentrations in vegans compared with all other groups. Also, iso-, anteiso-, and total BCFAs were much lower in vegans than in any other diet group. For *n*-6 PUFAs, we observed only one statistically significant difference between vegans, who had the highest level, and vegetarians, who had the lowest. In contrast, *n*-3 PUFAs levels were similar in vegans and vegetarians, and significantly lower than in pescatarians and omnivores. Substantial differences were found in EPA and DHA, where vegans and vegetarians were outstanding groups and had lower levels of these FAs than pescatarians and omnivores. Similar observations were made for DHA, with additional differences between both plant-based diet groups. LA levels were significantly lower in vegetarians compared with vegans and pescatarians, and omnivores had lower levels than vegans and pescatarians. For ALA, we found only one statistically significant difference between vegetarians and omnivores, where the former had lower concentrations of this FA (Table 2).

We also observed differences in FAs consumption, except for overall *n*-6 PUFAs and LA. In vegans, the LA intake seemed higher than in other groups, but it did not reach statistical significance (Table 3). Vegans consumed significantly fewer SFAs than all other groups. They also ate less MUFAs compared with pescatarians and omnivores, while having the highest total PUFAs intake, but for pescatarians, the difference did not reach statistical significance, though it was close to doing so. The total *n*-3 PUFAs intake was significantly higher in pescatarians than in all other groups. Surprisingly, there were no statistically significant differences between diet groups in *n*-6 PUFAs. The EPA and DHA intake was low in all analyzed groups, with the only significant difference found between vegans and omnivores, where the former group consumed these FAs in negligible amounts. ALA consumption was higher in pescatarians than in all other groups. The *n*-6/*n*-3 PUFAs ratio was the highest in vegans, and this result was statistically significant for omnivores (Table 3).

We observed differences in the intake of selected nutrients (Table 4). Vegans and vegetarians consumed less protein and less total fat than omnivores. Total carbohydrates and fiber consumption was higher in vegans than in vegetarians. Additionally, vegans ate more fiber than omnivores.

Analyzing all diet groups together, we found no correlation between the serum levels and consumption of selected FAs (SFAs, MUFAs, *n*-3 PUFAs, EPA + DHA, and ALA), except for *n*-6 PUFAs, for which there was a weak positive correlation (r = 0.25; *p* = 0.02; n = 89). Additionally, there was no statistically significant relationship between DHA serum levels and the consumption of ALA (its natural precursor) in any of the examined diets (Table 5).

**Figure 1:**
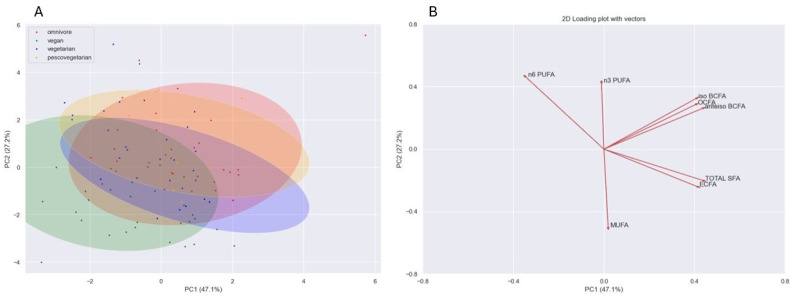
The results of principal component analysis (PCA) based on the serum levels of selected FAs. Scatterplot of individuals from different diet groups (**A**); variables (**B**).

**Figure 2:**
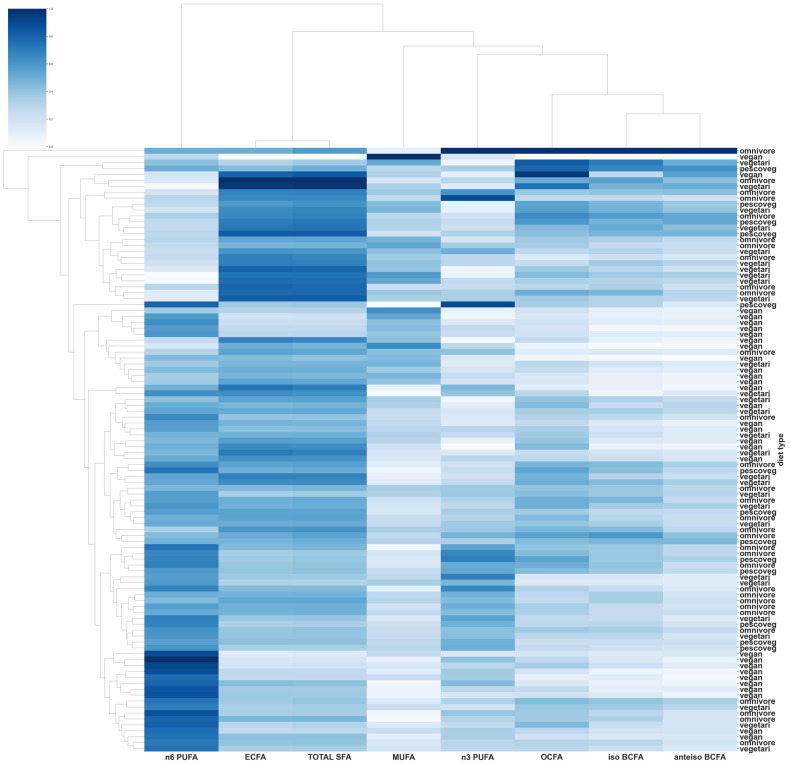
Heatmap generated based on the serum profiles of selected groups of FAs in individuals from different diet groups. Columns: fatty acids; rows: individuals. Color key indicates FA expression values: dark blue—highest; whitish—lowest.

PCA revealed a slight separation between vegans and the other diet groups, which is depicted in the scatterplot (Figure 1). A heatmap based on the FAs profiles, as a result of the hierarchical cluster analysis (HCA) of biomarkers, was used to assess the relatedness between different FAs and the visual presentation of the diversity of samples (Figure 2).

The median serum CRP levels were 0.01 ± 0.00 mg/dL in vegans, vegetarians, and pescatarians, and 0.01 ± 0.29 mg/dL in omnivores. We found that vegans had significantly lower levels of this protein than omnivores (H_3.79_ = 28.87; *p* = 0.04), while there were no differences between omnivores and pescatarians (*p* = 0.26). The difference in serum CRP levels between omnivores and vegetarians was close to reaching significance (*p* = 0.06).

## 4. Discussion

According to the vast amount of literature, plant-based diets provide health benefits, associated, inter alia, with a lower intake of SFAs, along with a higher consumption of PUFAs [8,9]. In our study, vegans had lower serum levels of SFAs compared with vegetarians and omnivores, and a lower SFAs dietary intake than any other diet group. Thus, the absence of dairy and meat (especially beef) in their diets, which are the main sources of these FAs, may have a cardioprotective effect, as they are known to increase cardiovascular risk [10]. 

On the one hand, as described above, a feeding pattern based solely on plant products is perceived as anti-inflammatory and cardioprotective [8,9]. On the other hand, however, a lower intake of crucial *n*-3 PUFAs (namely, EPA and DHA), as observed in vegan individuals, may carry the risk of negative effects on the cardiovascular system [11]. Nevertheless, there is a consistency in the literature indicating that different types of vegetarian diets have a lower cardiovascular risk compared with an omnivore diet [12]. We observed that vegetarians, especially vegans, had lower serum levels of EPA and DHA, which is in accordance with previous studies [13,14]. The dietary sources of these FAs are mainly marine fish, which are absent in a vegan diet. As EPA and DHA not only have cardioprotective and anti-inflammatory effects but are also crucial for brain development and function [15,16], it is recommended for vegans to supplement these nutrients [17]. Theoretically, vegans could take supplements with ALA or may increase their consumption of this essential FA, which is the precursor for the endogenous synthesis of EPA and DHA, from natural sources. It should be noted, though, that there is evidence that EPA and DHA supplementation is more effective than ALA supplementation, as the latter does not significantly boost the *n*-3 PUFAs desaturation pathway [18]. Generally, it seems that ALA supplementation is quite effective in increasing EPA levels, but has little or no effect on DHA, as summarized by Brenna et al. [19]. This is in accordance with a study showing that neither the dietary LA-to-ALA ratio nor total LA and ALA intakes correlate with DHA status [20]. In this paper, we also observed no correlation between ALA intake and DHA status in any of the analyzed groups, which supports previously described results. On the other hand, supplementation with preformed EPA and DHA significantly raises their levels in vegetarians, including vegans [14,21]. 

Regardless of the diet type, the conversion of ALA into EPA and DHA has low efficacy, and the crucial enzyme involved in this pathway is delta-6-desaturase. Its activity is under the influence of numerous factors and may be decreased by aging, infections, or stress hormones [22]. Additionally, nutritional factors may also play a role. Both macro- and micronutrients have been shown to affect the activity of delta-6-desaturase. The desaturation pathway is suppressed mainly by a high-fat diet, essential fatty acid deficiency, a high cholesterol intake, a low total protein intake, a low methionine intake, a low carbohydrate intake, a high folic acid intake in the absence of B_12_, a low zinc intake (in humans), and iron deficiency (also in humans) [23]. While most research on this subject has been conducted on animals, some papers also pertain to humans. For instance, soy consumption suppressed the desaturation pathway in a population of young adults [24]. Therefore, as the efficacy of the desaturation pathway, especially DHA synthesis, is influenced by numerous diverse factors, including nutritional and other factors, it seems reasonable to recommend supplementation with EPA + DHA instead of ALA.

While vegans had the lowest serum levels of total *n*-3 PUFAs, they had the highest levels of LA, which is in accordance with other studies [25,26]. Surprisingly, we found no differences in LA intake between the analyzed groups, while most papers have documented a higher intake of this FA in vegetarians, especially vegans [27]. LA consumption tended to be higher in vegans than in the three other groups, but this result did not gain statistical significance. A similar observation was made for the dietary *n*-6/*n*-3 PUFAs ratio, which also tended to be higher in vegans than in any other diet group but reached significance only for omnivores. This may be explained by the higher intake of energy from fats in this group compared with vegans and vegetarians. This essential *n*-6 PUFAs is abundant in plant-based products commonly used by the general population, and sunflower oil (one of the most LA-rich oils) is the third most widely cultivated oilseed in the world [28]. When we look at the *n*-6/*n*-3 proportion, not the absolute amount of LA consumed, vegans indeed have a higher intake than omnivores. Moreover, there are other studies showing similar consumption of the aforementioned FAs in vegans and omnivores. Mann et al. [29] documented a comparable dietary consumption of LA in vegans, vegetarians, and high-meat eaters. In another study comparing nutrient intakes in vegans, vegetarians, flexitarians, and omnivores, the authors observed no statistically significant difference in LA consumption between all studied groups [30]. It seems that LA intake may vary considerably depending on the individual intake of plant foods, especially oils. Some authors propose the diversity of regional cuisines as a possible explanation for the observed discrepancies in LA intake in vegans. For instance, Mexican individuals after exposure to a vegan diet for at least 3 years had LA concentrations that were over two times lower than those in omnivores [6].

Branched-chain fatty acids (BCFAs) have molecules with at least one methyl branch in their carbon chains. It has been shown that these FAs are increased in subjects with obesity, both in serum [31] and in adipose tissue [32]. Our results show low BCFAs levels in vegans compared with every other diet group. Since BCFAs seem to have anti-inflammatory properties, such as EPA and DHA, one would expect adverse health effects, namely, inflammation, in such subjects. However, the analysis of serum CRP, which is one of the most valuable biomarkers of inflammation used for prognosing cardiovascular events [33], indicated no inflammation process in subjects following plant-based diets. The concentrations of this marker were similar between omnivores and both pescatarians and vegetarians and were even lower in vegans. This is in accordance with other studies, showing low levels of circulating CRP in vegans compared with omnivores [34]. The same meta-analysis of 21 cross-sectional studies revealed a lower level of CRP in vegetarians, though it was less pronounced. This is also in accordance with our results, as the lower CRP levels in vegetarians were close to reaching statistical significance. In another study assessing and comparing inflammatory statuses in healthy subjects following different diets, inflammatory biomarkers, including CRP, among others, were similar in vegans, vegetarians, and omnivores [35]. Therefore, inflammatory status is under the influence of factors other than solely dietary pattern, and lower BCFAs concentrations in individuals following different types of plant diets presumably do not introduce a higher risk of inflammation and associated failure of the circulatory system. In our study, vegans had low iso-BCFAs and CRP levels. The low total BCFAs levels in vegans may be due to a lack of dairy products in their diet, as they are the main exogenous source of these FAs [2]. OCFAs are also documented for their anti-inflammatory and cardioprotective properties. Dietary OCFAs, such as BCFAs, are derived mainly from dairy and meat of ruminants [36]. It is documented that a high OCFAs intake lowers circulating cholesterol [2]. However, vegans in our study had the lowest level of OCFAs, and it is well documented that users of this diet have lower LDL and total cholesterol levels [5,37]; thus, there seem to be no adverse effects of low concentrations of these FAs in vegans.

## 5. Conclusions

Our findings make a new contribution to the understanding of how vegan and vegetarian diets affect human health, which is relevant, as more and more individuals turn toward these eating patterns. We observed lower levels of OCFAs and BCFAs in vegans. Additionally, both vegans and vegetarians had lower total n-3 PUFAs, and EPA and DHA serum levels than omnivores. However, decreased levels of these FAs did not cause inflammation in vegetarians and vegans. In fact, vegans had significantly lower levels of CRP compared with omnivores, despite having low EPA and DHA statuses, suggesting that other factors have an impact on the inflammatory status and overall health of subjects on plant-based diets. It should be noted, however, that our study included only women, and there are some differences in lipid metabolism between sexes. Further studies on long-term vegans and vegetarians are needed to address this issue. Nevertheless, n-3 PUFAs supplementation would be a good option for these diet groups, preferably in the form of products with sea algae-based EPA and DHA, instead of ALA. 

## Figures and Tables

**Table 1:** Comparison of basic characteristics in different diet groups.

	Vegans (n = 30)	Vegetarians (n = 28)	Pescatarians (n = 13)	Omnivores (n = 31)	*p*
Age [y]	28.5 ± 6.3	27.2 ± 4.4	27.8 ± 7.7	35.6 ± 10.4 *	<0.001
Body mass [kg]	58.6 ± 6.1	62.4 ± 5.4	60.2 ± 6.4	61.1 ± 9.2	0.16
BMI [kg/m^2^]	21.0 ± 1.8	21.6 ± 1.7	22.0 ± 2.0	22.0 ± 1.9	0.19
Body fat [%]	24.8 ± 3.8	25.0 ± 3.8	24.5 ± 3.5	27.5 ± 6.2	0.08
WHR	0.77 ± 0.05	0.75 ± 0.04	0.76 ± 0.05	0.81 ± 0.04 *	<0.001

Data are shown as means ± standard deviation. BMI—body mass index; WHR—waist-to-hip ratio. * Group with significantly higher values of analyzed parameters than in all other groups.

**Table 2:** Serum levels of selected FAs in different diet groups.

FAs Levels [%]	Diet Type	Mean ± SD	n	*p*					
				Vgn vs. Vgt	Vgn vs. Psc	Vgn vs. Omv	Vgt vs. Psc	Vgt vs. Omv	Psc vs. Omv
ECSFAs	Vgn	31.51 ± 3.46	30	**0.004**	0.260	**0.030**	0.054	0.400	0.230
	Vgt	34.36 ± 3.34	28						
	Psc	32.52 ± 2.01	12						
	Omv	33.60 ± 2.16	30						
OCFAs	Vgn	0.55 ± 0.11	29	**<0.001**	**<0.001**	**<0.001**	**0.020**	0.490	0.090
	Vgt	0.68 ± 0.10	26						
	Psc	0.77 ± 0.16	13						
	Omv	0.71 ± 0.11	30						
Iso-BCFAs	Vgn	0.09 ± 0.03	28	**<0.001**	**<0.001**	**<0.001**	0.060	0.080	0.440
	Vgt	0.19 ± 0.06	27						
	Psc	0.22 ± 0.05	13						
	Omv	0.22 ± 0.06	30						
Anteiso-BCFAs	Vgn	0.09 ± 0.03	28	**<0.001**	**<0.001**	**<0.001**	**0.020**	0.990	**0.010**
	Vgt	0.18 ± 0.06	28						
	Psc	0.22 ± 0.08	13						
	Omv	0.17 ± 0.04	29						
Total BCFAs	Vgn	0.19 ± 0.06	28	**<0.001**	**<0.001**	**<0.001**	0.160	0.600	0.340
	Vgt	0.38 ± 0.11	27						
	Psc	0.47 ± 0.15	13						
	Omv	0.41 ± 0.10	30						
SFAs	Vgn	32.31 ± 3.57	30	**0.001**	0.110	**0.010**	0.080	0.440	0.280
	Vgt	35.48 ± 3.41	28						
	Psc	33.76 ± 2.17	12						
	Omv	34.76 ± 2.19	30						
MUFAs	Vgn	31.72 ± 3.42	29	0.830	0.150	0.230	0.110	0.180	0.750
	Vgt	31.92 ± 2.85	28						
	Psc	30.40 ± 2.37	13						
	Omv	30.67 ± 2.55	31						
*n*-3 PUFAs	Vgn	0.87 ± 0.37	30	0.310	**<0.001**	**<0.001**	**0.010**	**0.010**	1.000
	Vgt	1.00 ± 0.48	28						
	Psc	1.27 ± 0.41	12						
	Omv	1.35 ± 0.41	29						
*n*-6 PUFAs	Vgn	34.61 ± 4.60	30	**0.040**	0.560	0.200	0.110	0.360	0.430
	Vgt	31.59 ± 4.70	28						
	Psc	33.85 ± 3.59	13						
	Omv	32.80 ± 4.11	31						
EPA	Vgn	0.21 ± 0.10	30	0.660	**0.020**	**<0.001**	**0.040**	**0.002**	0.210
	Vgt	0.23 ± 0.11	27						
	Psc	0.30 ± 0.10	11						
	Omv	0.34 ± 0.12	30						
DHA	Vgn	0.22 ± 0.17	30	**0.050**	**<0.001**	**<0.001**	**<0.001**	**0.050**	0.120
	Vgt	0.48 ± 0.27	28						
	Psc	0.74 ± 0.36	13						
	Omv	0.63 ± 0.23	29						
LA	Vgn	31.12 ± 3.93	30	**0.020**	0.770	**0.030**	**0.040**	0.910	**0.040**
	Vgt	27.90 ± 0.39	28						
	Psc	30.73 ± 1.41	9						
	Omv	28.05 ± 3.99	31						
ARA	Vgn	2.54 ± 0.99	30	0.740	0.130	**<0.001**	0.210	**<0.001**	0.056
	Vgt	2.62 ± 0.98	28						
	Psc	3.04 ± 1.09	9						
	Omv	3.66 ± 0.90	31						
ALA	Vgn	0.15 ± 0.06	29	0.110	0.860	0.280	0.120	**0.010**	0.240
	Vgt	0.11 ± 0.06	27						
	Psc	0.14 ± 0.05	13						
	Omv	0.17 ± 0.08	30						

Data are shown as means ± standard deviation and analyzed using one-way ANOVA and Duncan’s test. Statistically significant results are bolded. FAs—fatty acids; SD—standard deviation; Vgn—vegan, Vgt—vegetarian, Psc—pescatarian, Omv—omnivore, ECSFAs—even-chained fatty acids, OCFAs—odd-chain fatty acids, BCFAs—branched-chain saturated fatty acids; Iso-BCFAs—iso-branched-chain saturated fatty acids; Anteiso-BCFAs—anteiso-branched-chain saturated fatty acids; SFAs—saturated fatty acids, MUFAs—monounsaturated fatty acids, *n*-3 PUFAs—*n*-3 polyunsaturated fatty acids, *n*-6 PUFAs—*n*-6 polyunsaturated fatty acids, EPA—eicosapentaenoic acid, DHA—docosahexaenoic acid, ALA—alpha-linolenic acid, LA—linoleic acid, and ARA—arachidonic acid.

**Table 3:** Daily intake of selected FAs in study groups.

FA Intake [g]	Diet Type	Mean ± SD	n	*p*					
				Vgn vs. Vgt	Vgn vs. Psc	Vgn vs. Omv	Vgt vs. Psc	Vgt vs. Omv	Psc vs. Omv
SFAs	Vgn	15.09 ± 7.05	30	**0.049**	**0.010**	**0.030**	0.430	0.760	0.600
	Vgt	19.26 ± 5.01	24						
	Psc	21.02 ± 8.19	12						
	Omv	19.92 ± 6.67	25						
MUFAs	Vgn	15.09 ± 7.05	30	0.070	**0.030**	**0.001**	0.600	0.110	0.250
	Vgt	19.06 ± 5.01	24						
	Psc	22.85 ± 6.51	11						
	Omv	25.19 ± 6.49	27						
Total PUFAs	Vgn	15.58 ± 7.33	30	**0.040**	0.080	**0.050**	0.610	0.820	0.750
	Vgt	11.75 ± 2.81	23						
	Psc	12.66 ± 4.16	11						
	Omv	12.14 ± 3.99	27						
*n*-3 PUFAs	Vgn	1.53 ± 0.98	28	0.570	**0.030**	0.770	**0.010**	0.420	**0.040**
	Vgt	1.33 ± 0.77	23						
	Psc	2.35 ± 1.44	11						
	Omv	1.63 ± 1.26	26						
*n*-6 PUFAs	Vgn	8.91 ± 5.11	27	0.380	0.880	0.120	0.340	0.430	0.100
	Vgt	7.62 ± 4.09	24						
	Psc	9.13 ± 3.01	11						
	Omv	6.44 ± 4.89	26						
EPA + DHA *	Vgn	0.00 ± 0.00	30	0.500	0.120	**0.001**	1.000	0.400	1.000
	Vgt	0.02 ± 0.04	23						
	Psc	0.03 ± 0.43	12						
	Omv	0.10 ± 0.19	24						
ALA	Vgn	1.73 ± 1.12	29	0.910	**0.004**	0.650	**0.010**	0.710	**0.010**
	Vgt	1.78 ± 1.05	24						
	Psc	3.00 ± 2.45	12						
	Omv	1.93 ± 0.91	27						
LA	Vgn	11.13 ± 6.27	30	0.280	0.270	0.260	0.920	0.880	0.950
	Vgt	9.58 ± 2.20	22						
	Psc	9.45 ± 2.93	10						
	Omv	9.36 ± 3.20	27						
*n*-6/*n*-3	Vgn	6.53 ± 2.42	26	0.120	0.060	**0.030**	0.670	0.450	0.700
	Vgt	5.30 ± 2.19	20						
	Psc	4.97 ± 1.67	8						
	Omv	4.67 ± 2.02	26						

Data are shown as means ± standard deviation and were analyzed using one-way ANOVA and Duncan’s test, except for EPA + DHA (*), for which presented values are medians ± interquartile ranges and were analyzed using Kruskal–Wallis’s test due to incompliance of the data with a normal distribution. Statistically significant results are bolded. FA—fatty acid; SD—standard deviation, Vgn—vegan, Vgt—vegetarian, Psc—pescatarian, Omv—omnivore, SFAs—saturated fatty acids, MUFAs—monounsaturated fatty acids, *n*-3 PUFAs—*n*-3 polyunsaturated fatty acids, *n*-6 PUFAs—*n*-6 polyunsaturated fatty acids, EPA—eicosapentaenoic acid, DHA—docosahexaenoic acid, ALA—alpha-linolenic acid, and LA—linoleic acid.

**Table 4:** Daily intake of selected nutrients in different diet groups.

Nutrient Intake	Diet Type	Me ± IQR	Mean ± SD	n	*p*					
					Vgn vs. Vgt	Vgn vs. Psc	Vgn vs. Omv	Vgt vs. Psc	Vgt vs. Omv	Psc vs. Omv
Protein [% of energy]	Vgn	11.3 ± 2.5		29	1.00	0.15	**<0.001**	0.47	**<0.001**	0.09
	Vgt	12.1 ± 11.9		25						
	Psc	13.9 ± 5.2		12						
	Omv	19.0 ± 11.1		27						
Fats [% of energy]	Vgn	30.0 ± 8.7		29	1.00	1.00	**0.006**	1.00	**<0.001**	0.38
	Vgt	28.5 ± 26.0		25						
	Psc	30.3 ± 4.8		11						
	Omv	35.0 ± 6.6		27						
Carbohydrates [% of energy]	Vgn	56.4 ± 6.5		27	**<0.001**	0.15	0.18	1.00	0.29	1.00
	Vgt	49.0 ± 46.7		25						
	Pcs	50.9 ± 6.9		12						
	Omv	51.8 ± 12.4		26						
Fiber [g]	Vgn		44.6 ± 15.7	30	**<0.001**	0.32	**0.03**	0.49	0.60	0.97
	Vgt		28.3 ± 10.7	24						
	Psc		36.6 ± 9.6	12						
	Omv		34.5 ± 13.0	27						
Total energy [kcal]	Vgn		2007 ± 506	30	0.74	0.97	0.91	0.99	0.98	1.00
	Vgt		1896 ± 385	24						
	Psc		1944 ± 257	11						
	Omv		1938 ± 304	27						

Presented values were analyzed using one-way ANOVA (fiber and total energy intake) or Kruskal–Wallis’s test (percentage of energy from macronutrients). Statistically significant results are bolded. Me—median, IQR—interquartile range, SD—standard deviation, Vgn—vegan, Vgt—vegetarian, Psc—pescatarian, and Omv—omnivore.

**Table 5:** Correlation between ALA intake and DHA serum levels.

Diet Type	r	*p*	n
Vegan	0.07	0.71	28
Vegetarian	−0.29	0.16	24
Pescatarian	−0.20	0.53	12
Omnivore	0.19	0.36	25

r—Pearson’s correlation coefficient.

## Data Availability

The datasets used and analyzed in the current study are available from the corresponding author on request.
